# Implementation of mHealth to support cancer diagnosis in Sub-Saharan Africa: A systematic review

**DOI:** 10.4102/phcfm.v17i1.4683

**Published:** 2025-03-05

**Authors:** Kirsten D. Arendse, Grace A. Baby, Teffanie Maramba, Jennifer Moodley, Fiona M. Walter, Suzanne E. Scott

**Affiliations:** 1 Centre for Cancer Screening, Prevention and Early Diagnosis, Wolfson Institute of Population Health, Queen Mary University of London, London, United Kingdom; 2 Cancer Research Initiative, Faculty of Health Sciences, University of Cape Town, Cape Town, South Africa; 3 School of Public Health, Faculty of Health Sciences, University of Cape Town, Cape Town, South Africa; 4 The Primary Care Unit, Department of Public Health and Primary Care, University of Cambridge, Cambridge, United Kingdom

**Keywords:** digital health, cancer, early diagnosis, mobile health, primary care, sub-Saharan Africa

## Abstract

**Background:**

A reduction in communicable diseases in sub-Saharan Africa (SSA) over recent decades has led to an increased life expectancy and non-communicable diseases such as cancer. However, cancer services in SSA remain inadequate. With increasing mobile use, mobile health (mHealth) has the potential to expand healthcare access.

**Aim:**

This systematic review aims to synthesise literature reporting on barriers and facilitators to the implementation and use of mHealth tools by patients or the public to support symptomatic cancer diagnosis in SSA.

**Method:**

A comprehensive literature search was conducted following the Preferred Reporting Items for Systematic Reviews and Meta-Analyses. Two researchers independently conducted title and abstract screening, full-text review and data extraction. Extraction templates were compared and data were synthesised. Quality was assessed using the Mixed Methods Appraisal Tool.

**Results:**

Of 7695 records identified, three quantitative and two mixed-methods studies were included, published between 2016 and 2022. The studies focused on Kaposi’s sarcoma, cervical cancer, breast cancer and any cancer. Three inter-related themes describe the barriers and facilitators: (1) user or population-related factors including access to mobile devices and connectivity, and language literacy; (2) mHealth tool-related factors such as tool accessibility and language translation; and (3) structural, societal or systemic factors such as sociocultural significance and stigma.

**Conclusion:**

Although SSA countries experienced similar challenges to mHealth tool use as high-income nations, some barriers such as limited mobile devices and connectivity were more severely evident.

**Contribution:**

The study findings can be used to guide future mHealth tool design and implementation strategies that are relevant to SSA.

## Introduction

With the global increase in digital technology use, digital health is rapidly expanding and has the potential to improve healthcare delivery worldwide. Electronic health or eHealth is defined by the World Health Organization as ‘the use of information and communication technology in support of health and health-related fields’ (Page ix).^1^ Mobile Health (mHealth), a subset of eHealth, encompasses the use of mobile or wireless devices such as phones, tablets and patient monitoring devices for medical and public health practice.^1^ Access to health in many resource-constrained countries in sub-Saharan Africa (SSA) is limited, particularly among rural communities, where people travel far distances to access health services.^2^ With the rapid increase in public use of mobile devices^3^ and expanding Internet access,^4,5^ mHealth has the potential to broaden the reach of healthcare and information access to remote communities, particularly with the rise in demand for digital technologies among younger generations^6^ and the young age distribution in the region, with a median age of 19 years in SSA.^7,8^ There has been a sustained growth of mobile use in SSA, that was accelerated by the coronavirus disease 2019 pandemic when mobile devices became the primary mode of connectivity. This fast-tracked the development and adoption of many digital- and mHealth tools,^6,9^ such as the use of telephone or video consultations (‘telemedicine’), text-based communication between health care workers (HCWs) and patients, and healthcare helplines.^10^

With the reduction in communicable diseases and subsequent increase in life expectancy in SSA, over the past few decades, has led to an increase in non-communicable disease^11,12^ including a doubling of cancer incidence over the past 30 years.^13^ However, cancer health services in the region have remained underfunded and insufficient.^14^ Despite public health interventions significantly reducing breast- and cervical cancer burden in high-income countries, SSA continues to suffer a high disease burden, with breast- and cervical cancer being the two leading causes of cancer mortality among women.^15^ The high cancer morbidity and mortality in SSA could be underpinned by economic and resource limitations, such as the lack of effective screening programmes, trained healthcare workers and facilities for cancer diagnosis and treatment.^16^ In addition, sociocultural challenges act as barriers to help-seeking and timely diagnosis, such as harmful misconceptions about cancer and treatment.^17^ The high proportion of people diagnosed with cancer at an advanced stage leaves few and more costly treatment options with poorer prognoses.^16^

Mobile health has the potential to support more timely diagnosis of cancer in symptomatic patients through widespread communication of health information to the population of SSA, of which nearly half (43%) subscribe to mobile services.^6^ Increasing public awareness and recognition of possible cancer symptoms could encourage earlier appraisal and help-seeking,^18,19^ reducing the time from first experiencing symptoms to diagnosis. A systematic review of digital technologies for cervical cancer control demonstrated that using digital health technologies (including short messaging services [SMS], podcasts, and telephone interventions) in low- and middle-income countries increased knowledge and awareness of cervical cancer.^20^ However, many studies in this review noted implementation challenges such as poor technological infrastructure and inequitable access to technology. While mHealth tool use in SSA is on the rise, there is little evidence demonstrating successful implementation and long-term sustainability.^21^ Implementing contextually suitable and financially viable initiatives to advance early cancer diagnosis has proven challenging in many settings in SSA.^22^ In this study, we aim to review the existing literature to identify and synthesise evidence on the barriers and facilitators to mHealth tool use by patients or the public to support symptomatic cancer diagnosis in SSA. This understanding could guide the development of sustainable and contextually relevant initiatives tailored to the needs of different communities.

## Methods

### Study design

This systematic literature review followed the Preferred Reporting Items for Systematic Reviews and Meta-Analyses guidelines and checklist.^23^ The review protocol was registered on The International Prospective Register of Systematic Reviews (PROSPERO) to allow for transparent reporting, ensure alignment with the research protocol and prevent duplication of work (reference CRD42024503906).^24^

### Search strategy and data sources

An initial literature search, guided by the research question and study inclusion criteria, was conducted on Google Scholar to identify key articles. Thereafter, the strategy was created with the help of a health sciences subject librarian and followed the Cochrane guidelines for searching and selecting studies for systematic reviews^25^; this included pre-testing the search strategy before the final search. MEDLINE (via PubMed), Cochrane, Scopus, Web of Science and CINAHL were searched using synonyms and descriptive headings, such as MeSH (medical subject headings) terms. The search was conducted from 15 June 2023 to 16 August 2023. The full search strategy is included in the registered protocol.^24^

### Eligibility criteria

Study inclusion and exclusion criteria were defined based on the Population, Intervention, Comparison and Outcome(s) Framework (Box 1).^26^ Primary studies were included if they were conducted within any SSA country, reported on the implementation or use of mHealth tools by patients or the public, and the tools assessed addressed key factors leading up to symptomatic cancer diagnosis within the ‘appraisal interval’ or the ‘help-seeking interval’ as described in the Model of Pathways to Treatment.^18,19^ The ‘appraisal interval’ is the time from experiencing bodily changes to perceiving a reason to discuss the symptom with a healthcare professional and the ‘help-seeking interval’ is the time from perceiving a reason to discuss the symptom with a healthcare professional to first consultation. Interventions that were considered to address these key areas leading up to symptomatic cancer diagnosis were predetermined and included in the protocol. This included the following interventions, mHealth tools providing support with: (1) awareness of symptoms and/or risk factors; (2) help-seeking behaviours; (3) self-examination; (4) healthcare utilisation; (5) attitudes to healthcare; and (6) access to healthcare. This includes studies regardless of whether the participants had symptoms, but where the tool aimed to address individual’s symptom awareness and health behaviour in response to symptoms if they were to occur. There were no restrictions on the type of comparator or controls, language or date. Studies that focused on cancer screening or primary prevention (i.e., for use among asymptomatic individuals, such as for cervical cancer screening, HPV [human papillomavirus] vaccination or smoking cessation) were excluded. Studies that sampled a specific sub-population of patients were excluded, for example, women with a positive HPV screening test or people with a known diagnosis of cancer.

BOX 1 Study inclusion criteria using the population, intervention, comparison and outcome(s) framework.

**Table:** 

**Inclusion criteria**	
Population	Studies conducted in any country in sub-Saharan Africa Interventions that have the potential to target individuals with symptoms of possible cancer (e.g., increasing awareness or changing behaviour in response to symptoms) Interventions designed for patient or public use
Intervention	Studies reporting on mHealth interventions
Comparator	No restriction on comparators or controls
Outcome(s)	Studies evaluating elements of the ‘appraisal interval’ or ‘help-seeking interval’ or factors that influence them

*Source*: McKenzie JE, Brennan SE, Ryan RE, Thomson HJ, Johnston RV, Thomas J. Chapter 3: Defining the criteria for including studies and how they will be grouped for the synthesis [homepage on the Internet]. [cited 2024 Jun 7]. Available from: https://training.cochrane.org/handbook/current/chapter-03

mHealth, mobile health.

### Study selection

Titles and abstracts of all identified publications were independently screened by two reviewers (G.A.B. and T.M.) to determine potential relevance. Each article identified during this first stage of screening underwent independent full-text review by K.D.A. and G.A.B. to determine selection based on the eligibility criteria. Disagreements were discussed and settled in consultation with K.D.A., G.A.B., F.M.W. and S.E.S.

### Data extraction and analysis

Data extraction tools were developed following guidelines from the Cochrane Handbook,^27^ and adapted from Nnaji et al.^22^ in consultation with the wider study team (G.A.B., F.W.M., S.E.S.). Extraction templates included six domains: (1) study identification details (title, journal, authors, country, language, year of publication); (2) study methodology (design, objectives, aims, research question, sample population); (3) participant characteristics (size, target group, sex, age, ethnicity, education, income); (4) mHealth tool details (type of tool and function); (5) study findings (reported outcomes, barriers and facilitators); and (6) conclusions. Two reviewers (K.D.A. and N.T.) independently extracted data, and K.D.A. compared extraction templates to check accuracy and completeness. Discrepancies in data extraction were discussed between reviewers. Findings were checked by F.M.W. and S.E.S. to ensure alignment with the study protocol. K.D.A. used the extraction templates to conduct the data synthesis and analysis.

### Quality assessment

Two reviewers (K.D.A., G.A.B.) independently assessed each included study for the risk of bias using the Mixed Methods Appraisal Tool (MMAT),^28^ an externally validated tool used to assess the risk of bias in mixed methods systematic reviews.^29^ The MMAT contains two screening questions and five varying criteria used to assess quality (Table 1-A1), based on the study type (qualitative, quantitative, randomised- or non-randomised control trials or mixed methods). The criteria can be *met* (‘Yes’) or *not met* (‘No’ or ‘Can’t tell’). Each criterion is assessed individually with no overall quality score. Disagreements between scores were resolved through discussion and consensus. Studies were included irrespective of quality scores.

## Review findings

### Search results

**FIGURE 1:**
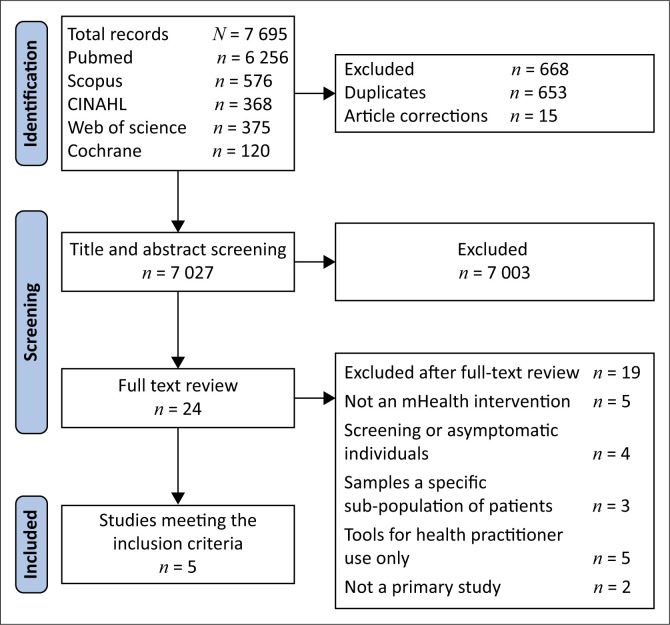
Flow diagram demonstrating the study search and selection process.

Across all databases, 7695 records were identified, of which 668 were removed (653 duplicates and 15 article corrections). Titles and abstracts were screened for the remaining 7027 unique records, of which 24 potentially relevant articles underwent full-text review. After applying the exclusion criteria (Figure 1), 5 studies were selected for inclusion.

### Characteristics of included studies

The five included studies (Table 1) were published between 2016 and 2022 across four SSA countries: Nigeria,^30^ Ghana,^31^ Malawi^32^ and Uganda.^33,34^ Two studies were conducted in urban setting,^30,31^ one in rural setting^32^ and two across both urban and rural settings.^33,34^ Two studies focused on cervical cancer,^31,32^ one on Kaposi’s Sarcoma,^33^ one on breast cancer^30^ and one on all cancer types.^34^ Two studies used a pre-and-post-intervention analysis,^32,33^ one was a descriptive observational study,^30^ and two used a mixed-methods design.^31,34^

**TABLE 1:** Overview of included studies, participant characteristics and the mobile health tool functions.

Author (year)	Country, setting	Target cancer type	Type of mHealth tool	Function of the tool	Type of mHealth tool engagement†	Communication between the tool user & study team	Participant characteristics	
							Sample population, sex, and age	Education
Salako et al.^30^	Nigeria, urban	Breast cancer	Telephone helpline: callers talked to live agents	Provided cancer information: screening, symptoms, self-examination, and where to access services	Interactive	Direct communication	Members of the general public who used the hotline (*N* = 294, aged 24–55 years): 285 (97%) female, 8 (27%) known with breast cancer	No education: 4 (1%) Primary: 8 (3%) Secondary: 36 (12%) Tertiary: 246 (84%)
Caster et al.^32^	Malawi, rural	Cervical cancer	An interactive audiovisual tool was shown to participants on a researcher-managed tablet	Provided cancer information: screening and risk factors	Interactive	Direct communication	Women (*N* = 243; aged 18–77 years) from 6 rural villages surrounding health centres, *n* = 126 (52%) women had previous knowledge of cervical cancer	No education: 51 (21%) Primary: 156 (64%) Secondary or higher: 36 (15%)
Bonful et al.^32^	Ghana, urban	Cervical cancer	Short messaging services (SMS) were sent to participants’ mobile devices	Provided cancer information: screening, risk factors, where to access health services	Passive	No direct communication	(1) 130 women, aged 13–39 years completed a survey, including 62 (48%) women with ‘white collar jobs’ (working in banks or the local municipality) and 68 (52%) from communities. From these women, 32 took part in focus group discussions; (3) stakeholder engagement with research and clinical experts (number not indicated); (4) Participants who received SMS (*n* = 8) gave feedback via interview	(1) Primary: 29 (22%) Secondary: 32 (25%) Tertiary: 64 (49%) (3)-(4) Not stated
Laker-Oketta et al.^33^	Uganda, urban (57%) and rural (43%)	Kaposi’s sarcoma	An educational video was shown on a researcher-managed tablet	Provided cancer information: symptoms, self-examination, risk factors, where to access health services	Passive	Direct communication	Members of the general public at markets aged > 18 years (*N* = 420; 210 [50%] were female; median age = 30 years)	Primary or no education: 70 (50%) Lower secondary: 46 (33%) Higher secondary or tertiary: 24 (17%)
Kabukye et al.^34^	Uganda, urban and rural	All cancer types	Telephone helpline: callers listened to pre-recorded audio messages and navigated the system using voice commands or dual-tone frequencies	Provided general information about cancer, address myths and answer questions	Interactive	No direct communication	(1) 40 tool users gave feedback via telephone survey (median age = 35 years); 14 (35%) female; in total, there were 3 820 interactions with the tool (calls made) from 1 230 unique phone numbers (2) Focus groups and interviews with the general public, healthcare providers, patients with cancer, caregivers and administrators (*N* = 73; 41 (56%) female, median age 37 years)	(1) Primary or no education: 13 (32%) Secondary:15 (37%) Tertiary: 12 (29%) (2) Primary or no education: 19 (26%) Secondary: 10 (14%) Tertiary: 44 (61%)

mHealth, mobile health; (1), group 1; (2), group 2; (3), group 3; (4), group 4.

† , Tools were considered interactive where users were required to take the initiative to engage with the material (e.g., call a telephone helpline or navigate a prompt on an mHealth tool by clicking a screen or using voice commands), whereas tools were considered passive where users passively received promotional material and engaged by watching, listening and/or reading material.

### Mobile health tool types and content

Tools identified in the studies were considered as those requiring ‘interactive’ or ‘passive’ user engagement (Table 1). Tools were ‘interactive’ if users were required to take the initiative to engage with it or respond through a physical action (such as making a call, speaking, typing or clicking). Tools were ‘passive’ if users were not required to take the initiative to use it (e.g., receive an SMS), and their engagement with the tool was non-physical or purely mental (e.g., reading an SMS or watching a video). Two studies used passive tools^31,33^ and three used interactive tools.^30,32,34^

Two studies provided mobile telephone helplines.^30,34^ Salako et al.^30^ provided a telephone helpline to which callers spoke directly to a person on the line, who provided information about breast cancer and answered questions. Kabukye et al.^34^ also provided a telephone service called an ‘interactive voice response’ system: users called the number, listened to a series of pre-recorded messages containing health information about different cancers and navigated menu options using voice commands or their mobile phone dialling pad (called ‘dual-tone messaging’). Users could request to be phoned by a person at a later time. Two studies showed participants educational videos on a tablet managed by a researcher.^32,33^ Caster et al.’s^32^ study used an interactive video about cervical cancer: participants actively engaged by clicking pop-ups on the tablet’s screen, and Laker-Oketta et al.’s^33^ tablet-based tool was an educational video about Kaposi’s sarcoma that participants passively watched and did not require interactive engagement. Bonful et al.^31^ provided educational messaging about cervical cancer using SMS: participants passively received messages with no interactive engagement with the tool.^31^ The tools provided general information about their targeted cancer type(s),^30,31,33,35^ cancer screening,^30,31,32^ cancer symptoms and self-examination,^30,33^ risk factors^31,32,33^ and where to access cancer health services.^30,33^ All five studies provided the tools in local language(s), and three additionally had options for English.^30,31,34^

### Study participant characteristics

The two telephone helplines^30,34^ were available to anyone who voluntarily called the advertised numbers without any noted limitation on age in the studies. The two mHealth interventions focusing on cervical cancer^31,32^ selected female participants only: for both the pilot testing and feedback interviews, Bonful et al.^31^ selected women aged 18 and 39 years (because of their interest in only evaluating women of reproductive age) who had never had cervical cancer or a cervical screening test, owned a mobile phone and were able to read SMS, whereas Caster et al.^32^ selected women of any age in the community surrounding a health clinic. Laker-Oketta et al.^33^ recruited participants from a local market. All studies reported on participant age, sex and education level, three^31,32,33^ reported on income and one on literacy level^33^ (Table 1). Three studies included participants with high levels of education with 49%,^31^ 50%^34^ and 84%^30^ of participants having tertiary education. In contrast, the two tablet-based educational video studies sampled participants who mostly had primary or no education at 85%^32^ and 50%.^33^

### Assessing study quality

Caster et al.^32^ and Laker-Oketta et al.’s^33^ whose studies had similar interventions and study designs, met the same four quality criteria (Table 1-A2). Both studies used unvalidated questionnaires, and reported change in questionnaire scores after the intervention without significance testing or an interpretation of the scoring system used. Therefore, it was unclear whether their measures were appropriate to evaluate outcomes (quality criteria 4.3).

The two telephone helplines^30,34^ and the SMS intervention^31^ did not have samples representative of their target population (quality criteria 4.2). Bonful et al.’s^31^ intervention to disseminate cervical cancer information was limited to women aged 18–39 years, excluding those above 39 years who would still be eligible for screening. In contrast, Caster et al.’s^32^ study included women of all ages, and older women felt it was inappropriate to teach them about cervical cancer because they were too old and not sexually active. Bonful et al.^31^ selected women working in banks or the local municipality to represent a higher socioeconomic group and women ‘in the community’ to represent a lower socioeconomic group. This was assumed and could have been measured by including a question on income in the survey. Salako et al.^30^ did not specify their target population. Despite limitations in Bonful et al.’s^31^ sampling strategy, the study had clear research questions, used appropriate methods to measure outcomes, and adequately synthesised findings.

Both Salako et al.^30^ and Kabukye et al.’s^34^ studies had insufficient information reported in their studies, unclear research questions and recruitment strategies with a high risk of bias. Both recruited participants through media campaigns, where participants voluntarily called a number, limiting participation to those with media and mobile phone access, network coverage and mobile credit. Salako et al.’s^30^ study was further limited to those available during working hours. Both studies conducted feedback surveys from a sample of callers, restricting participation to those who answered their phones. Salako et al.’s study had no explicit research question or aims; however, presumed from the text (to describe the implementation of the intervention and measure behavioural and clinical outcomes among callers), the data collection strategies would be appropriate. Kabukye et al.^34^ used appropriate measures to assess cancer awareness, which was appropriately interpreted and discussed. However, it is unclear how they measured acceptance and user experience. The results of the questionnaires/surveys were not published and they did not report how they analysed or synthesised the quantitative and qualitative findings (MMAT criterion 4.3–4.5).

### Barriers and facilitators to tool use and implementation

Study outcomes that directly acted as barriers or facilitators to tool use or implementation are reported in Table 2 and Table 3. Three inter-related themes were developed to describe the reported barriers and facilitators; this included: (1) user and population-related factors; (2) mHealth tool-related factors; and (3) structural, societal and systemic factors. User and population-related factors included participant or population characteristics, and individuals’ access to resources that impacted their ability to use mHealth tools. mHealth tool-related factors included elements of tool design and implementation strategies that determined its usability, accessibility and reach. Structural, societal and systemic barriers and facilitators were factors that impacted the broader population and had the potential to influence health beliefs and help-seeking behaviours. Some factors may have fit into more than one theme, but were discussed under the theme in which a stronger relation was determined or where outcomes were more strongly reported in the studies. For example, some user and population factors or structural, societal and systemic factors were also considered to be mHealth tool-related if there were tool design considerations (or a lack thereof) specific to the population that used the tools, such as adaptations for population characteristics or sociocultural norms.

**TABLE 2:** Overview of the barriers and facilitators to mobile health tool use and implementation measured in the included studies.

Main theme	Sub-theme	Salako et al.^30^	Caster et al.^32^	Bonful et al.^31^	Laker-Oketta et al.^33^	Kabukye et al.^34^
User or population-related factors	Access to personal devices or network	X	N/A – device provided	X	N/A – device provided	X
	Language literacy	-	X	X	X	X
	Tech literacy	-	X	-	X	-
mHealth tool-related factors	Pilot user feedback and experience	-	-	X	-	X
	Accessibility (ease of use, timing & location)	-	-	X	-	X
	Prospective requirements for design amendments	-	-	X	-	X
	Relevance, attitude, and acceptability	-	X	X	-	X
	User uptake and utilisation	X	X	X	-	X
Structural, societal or systemic factors	Access to health services	-	X	-	-	X
	Socio-cultural beliefs and significance	-	-	X	-	-

mHealth, mobile health; N/A, not applicable.

**TABLE 3:** Barriers and facilitators to mobile health tool use and implementation reported in the included studies.

Main theme	Sub-theme	Barriers	Facilitators
User or population-related factors	Access to mobile devices or network	Lack of access to mobile phones^31,34^	Widespread access to mobile phones^30^
		Limited access to data or network^31^	
	Language literacy	Poor language literacy^31,33,34^	
	Tech literacy	Little or no previous experience with technology^32^	Previous experience with technology^34^
mHealth tool-related factors	Pilot user feedback and experience	Messages were too frequent^31^	Tool was easy to use^31^
		Messages were repetitive^31^	-
		Messages were irritating^31^	-
		Messages were too long^31^	-
		Messages or prompts were confusing^31,34^	-
		Messages were uninteresting^31^	-
		Messages without information (questions only)^31^	-
		No direct engagement with the study team (unable to interact or ask questions)^31,34^	-
		Users required assistance from study team to use the tool^32,33,34^	-
		Mobile battery power drained by tool^31^	-
	Accessibility (ease of use, timing & location)	Only available during working hours^30,31^	Tool available at all hours^34^
		Cost to participants^30,31^	Free^34^
		Poor automated translation of local language^34^	Tool available in local language^33^
		Unable to recruit participants from rural areas^30^	Able to participate from any location^34^
		Participants did not answer phones after a call back request^34^	Able to listen to messages repeatedly^34^
	Prospective requirements for design amendments	-	Use of bold text in messages^31^
		-	Receipt of messages outside working hours^31^
		-	Use of catchy phrases and hashtags^31^
		-	Novel information (not repetitive)^31^
		-	Short messages^31^
		-	Infrequent messages^31^
		-	Use of celebrities, professionals or cancer survivors to campaign for tool or service uptake^31,34^
	Relevance, attitude, and acceptability	Agents on helplines unable to offer medical advice, only health education and invite participants for screening^30^	Learning from a tablet was preferrable to learning from a person^32^
			Positive attitude and acceptance towards tools/technology^31,32,34^
		Tools were inappropriate for the target age group or generally for public consumption^32,33^	
			Interest in the future use of mHealth tools^31,34^
	User uptake and utilisation	Poor user uptake^30^	-
		Tools that required participants to take an active role and initiative to use the tool^30,34^	
Structural, societal or systemic factors	Access to health services	No link to free health services being promoted by the tool^31^	-
	Socio-cultural beliefs and significance	Harmful myths and misconceptions about cancer^34^	Engagement was higher in a village with the recent death of a woman with cervical cancer^32^
		Stigma towards people with possible cancer^34^	

mHealth, mobile health.

#### User and population-related factors

User and population-related barriers included low language literacy^31,33,34^ and poor mobile network coverage.^31^ The lack of previous experience with technology was reported as a barrier in one study^32^ and experience with technology was reported as a facilitator in another.^34^ Two studies reported limited access to personal mobile phones as a barrier,^31,34^ whereas one study reported high availability of personal mobile phones (90%) as a facilitator.^30^ In three studies, participants were required to have access to a personal or borrowed mobile device to participate, whereas the remaining two studies provided the mobile devices used by participants.^32,33^

#### Mobile health tool-related factors

A barrier to engagement in two studies was the cost they incurred on users,^30,31^ whereas one reported the helpline being free as a facilitator.^34^ Two studies reported that availability during work hours only restricted use,^30,31^ whereas in Kabukye et al.’s study,^34^ the telephone helpline was available at all hours, facilitating its use. In one study, participants complained that reading SMS drained their mobile battery.^31^ While this was reported as a barrier related to the mHealth tool, it may be considered a user or population barrier (e.g., limited access to mobile battery, poor mobile battery capacity, or a lack of electricity) or a systemic factor (e.g., poor electrical infrastructure); however, none of these was explicitly described in the studies.

In three studies, participants required assistance using tools from the study team^32,33^ or family members,^31^ although one of these studies^32^ also reported that participants found the tool ‘easy to use’. Users disliked messages that were frequent, long, repetitive or asked questions rather than provided information, and some reported that messages or prompts were uninteresting^31^ or confusing.^31,34^ In the feedback for two studies, participants suggesting using important people to campaign for uptake, such as endorsement by celebrities, professionals or cancer survivors.^31,34^

Participants reported that taking the initiative to make a call in the two telephone helpline studies restricted engagement and users suggested in feedback that the researchers actively call participants.^30,34^ Participants using tools without direct communication with researchers or live agents reported difficulty in not being able to ask questions, interact or ask for assistance.^31,34^ One study allowing for direct engagement with active agents through the helpline noted a limitation in the agents’ skills: they were unable to provide medical advice, only education and invitations for screening.^30^

There were positive attitudes towards the tools across three studies,^31,32,34^ with one reporting a preference for learning from a tablet rather than a person,^32^ and two reporting an interest in future engagement in healthcare activities using mHealth.^31,34^ Yet two studies reported the tool was inappropriate: in Caster et al.’s^32^ study on cervical cancer education, elderly women felt that they were not at risk because of their age and not being sexually active, and Laker-Oketta et al.’s^33^ study reported that the tool was ‘inappropriate for public consumption’ without mention of what this meant. Two studies measured tool uptake after a period of media campaigns to promote use,^30,34^ although only one reported utilisation as poor compared to expected participation particularly among rural users.^30^

#### Structural, societal and systemic factors

Few structural, societal and systemic barriers and facilitators were reported in the studies, despite a significant focus in their rationales. In Bonful et al.’s^31^ feedback surveys, participants noted disinterest in the campaign because they did not directly offer linkage to health services that were being promoted by the tool, rather, they provided advice on where to access services.^31^ In Caster et al.’s^32^ study, engagement with the cervical cancer educational video tool was greater in a village where someone recently died of the disease and sparked an interest in cervical cancer in the local community. Kabukye et al.^34^ reported harmful myths and misconceptions about cancer: some participants believed cancer is caused by witchcraft, that biopsies lead to rapid disease progression and that cancer is transmittable. Participants reported being discouraged by relatives and caregivers from seeking formal cancer services and opted to use traditional medicine as an alternative,^34^ which could extend to acting as a barrier to cancer health services, including the use of mHealth tools for possible symptoms of cancer. Poor mobile network coverage was also considered a structural barrier in addition to being user and population-related.

## Implications and recommendations

Key recommendations were developed to guide future design of mHealth tools and important considerations for implementation strategies. These recommendations were developed based on the synthesis of barriers and facilitators identified from the five studies included in this review (Box 2).

BOX 2 Key recommendations to develop and implement mobile health tools for use in sub-Saharan Africa among the public to support symptomatic cancer diagnosis.

**Table:** 

**Recommendations for mHealth tool design** Tools should be available in local languages Messages should be kept short Messages should contain novel information (in comparison to only asking questions or providing repeated information) Tools should allow two-way communication or direct communication with the service provider Tools should be free to participants Tools should minimise mobile battery power usage Tools should minimise the need for data and mobile connectivity, or be usable offline Tools should be available on analogue mobile devices where possible (i.e., can be used without Internet access) Tools should preferably use media accessible to users with limited written literacy or visual impairment (e.g., audio-visual media preferable to text)
**Implementation and contextual considerations** Community and patient consultation in tool and implementation design Endorsement from public figures, celebrities or cancer survivors to support uptake and campaigning Active recruitment of participants Availability of free health care services promoted by the tool should be a key consideration in the investment, design and implementation, and should support linkage or direct services where possible Campaigns to address stigma around cancer and cancer health services should be conducted alongside strategies to support tool uptake Expand internet access, such as advocating for free access to public internet hotspots

mHealth, mobile health.

### Discussion

Previous literature reviews evaluated the barriers and facilitators to digital technologies use among healthcare professionals,^36,37^ for non-cancer related-health conditions^38,39^ and in high-income countries.^38,39,40^ This systematic review is the first to synthesise literature to report on the barriers and facilitators to the implementation and use of mHealth tools by patients or the public to support symptomatic cancer diagnosis in SSA. Our review shows sparse literature exploring the topic, with only five studies eligible for inclusion after thoroughly searching several databases. All five studies had bias and design limitations, demonstrating the lack of quality literature and therefore, reliable evidence to guide development and implementation of similar interventions in the region. Nevertheless, the included studies provide useful lessons, including public perspectives on important factors to include in future mHealth tool design and implementation.

The positive attitudes towards mHealth tools reported in three studies^31,32,34^ evaluated in this review suggest a reassuring landscape for potential expansion in SSA. Tool designers should note aspects of digital text that are facilitators to engagement, such as short, emboldened, catchy and novel phrases, and those that act as barriers, including frequent, repetitive messages or messages asking questions, which was similarly found in another review.^41^ Furthermore, limiting the need for participants to take the initiative to engage with tools could increase uptake, such as through actively calling people, sending SMSs, or advertisements through mobile applications using text, audio or visual media.

Similar to another review conducted predominantly in high-income countries,^38^ user- and population-related barriers to mHealth tool use were common, including poor data and network connectivity.^31,34^ Many countries in SSA have unreliable sources of electricity and lack access to mobile network coverage and the Internet, making mHealth use challenging,^42^ particularly in rural areas where the majority of the population resides.^43^ Even in countries with rapidly growing technological advancements such as in South Africa, the adoption of digital health tools is severely limited by poor mobile connectivity in rural areas,^44^ frequent power cuts and a lack of electrical infrastructure.^45^ To overcome challenges with internet connectivity, designers should consider developing tools that can be used offline or on mobile devices without internet access. In a review exploring digital health use globally, access to free Internet or public hotspots were facilitators to engagement with digital health tools^41^ and should be considered to improve internet access and support mHealth tool expansion in SSA. Access to mHealth could also be increased through using automated systems such as in Kabukye et al.’s study,^34^ because of their lack of need for live agents and potentially lower cost. With rural communities being restricted by far distances and costly travel expenses to access healthcare services,^2^ accessing health information at an individual’s convenience through a mobile device has the potential to limit unnecessary, costly healthcare visits and increase the uptake of services among individuals who would otherwise have not sought medical care for symptoms of possible cancer. In one study,^31^ users felt disinterested in a tool because it promoted a service without direct linkage to free health services or screening. This highlights a challenging ethical concern of promoting healthcare in resource-limited areas where the services promoted are either not accessible or incur a financial or social cost to individuals. Mobile health tool developers and researchers should adequately invest in understanding the landscape of access to local cancer health services in the communities they plan to serve before design or implementation.

Accessibility of tools was reported in several studies, noting that availability in local languages^33^ and being free of charge^34^ were facilitators to use. In Kabukye et al.’s^34^ study, the tool was translated into local languages; however, participants struggled to understand the audio messages and the tool had difficulty capturing participants’ voice commands. The poor integration of local languages into voice recognition software highlights an important area for future investment in research and development. The use of audio and/or visual tools as used in four^30,32,33,34^ of the five studies has the potential to widen accessibility to participants with limited reading and/or written language literacy or visual impairment, compared to tools using digital text, such as in Bonful et al.’s study,^31^ which limits accessibility to literate participants. While design considerations should be made to maximise the ease of tool use, it is equally important to create campaigns for uptake using media accessible to a broader selection of the population, accounting for varying education levels, literacy, prior experience with mobile technologies, geographical location and access to mobile devices.

All studies in this review restricted participation to individuals with high levels of education directly, or indirectly by mobile literacy being a prerequisite for inclusion, and thus samples were not representative of the target populations. In Kabukye et al.’s^34^ automated telephone helpline, users had the option to request a call from a live agent; however, users were frequently uncontactable on attempts to phone them back, which may be because of the use of borrowed devices, lack of mobile battery or users not recognising the number calling them. In Salako et al.’s^30^ helpline, users reported the limited medical knowledge of the agents; participants may have expected the service to provide or replace routine healthcare services, and caution should be made in creating false expectations among the public as to the role of these interventions through community participation in development and awareness campaigning.

Previous literature has demonstrated that sociocultural norms and beliefs in SSA can delay help-seeking, such as the belief that people with cancer are cursed or being punished by ancestors,^46,47,48,49^ a preference for traditional, complementary or alternative medicines,^50^ requiring family or spousal permission to access cancer or screening services,^48,50^ and being discouraged by communities from seeking Westernised biomedical cancer services.^50^ This was similarly noted by Salako et al.,^30^ and the low uptake of the widely advertised tool may be explained by the stigmatising beliefs about cancer health services leading to apprehensive health behaviours. Two studies^31,32^ highlighted the importance of sociocultural norms in cancer health campaigns, demonstrated by users recommending the involvement of local celebrities, professionals or cancer survivors in campaigns, as well as by the greater engagement among participants in a village that had a recent death because of cervical cancer.^32^ Thus, the success and sustainability of mHealth interventions for cancer care may be dependent on coexisting strategies to address sociocultural beliefs about cancer, and the involvement of local communities in the design of tools and implementation strategies. The participation of patient and community representatives in tool design can also limit the use of culturally inappropriate content,^33^ prevent the recruitment of target age groups that are too narrow or wide,^31,32^ or using the wrong target group altogether,^30^ as seen in the studies evaluated in this review.

### Strengths and limitations

This review used a systematic approach to thoroughly search for relevant literature using multiple databases and specifically focused on SSA to identify barriers and facilitators contextually relevant to this region. The inclusion of quantitative, qualitative and mixed methods studies broadened the scope of potential studies for inclusion. The protocol was published on PROSPERO to ensure the study processes aligned with planned procedures with transparency and to prevent duplication of work. The study offers a novel perspective to the limited body of literature on mHealth interventions to support symptomatic cancer diagnosis in SSA. The small number of eligible studies demonstrates the need for further research. With few studies included in this review and marked heterogeneity, it was difficult to make comparisons between them.

All five studies had bias, with the unmet quality criteria being most relevant to inadequately reported study outcomes. Because the barriers and facilitators in the included studies were reported through user feedback and experience, rather than as outcomes, there was still value in synthesising findings despite their limitations in quality. Restricting this review to studies in SSA limited the opportunity to explore literature in other low-resourced regions and may have provided additional insights. Despite the few studies and low quality, we were able to synthesise important lessons that can be used to guide mHealth tool development and implementation strategies to support symptomatic cancer diagnosis in SSA.

## Conclusion

With the growing use of mobile devices in SSA and the lack of access to quality healthcare and information in the region, mHealth has the potential to enhance access, particularly to rural communities. This review demonstrates the lack of quality literature evaluating mHealth tools to support symptomatic cancer diagnosis in SSA, in contrast to other literature showing widespread use of digital tools among healthcare workers, integration into digital health systems, and for use among asymptomatic people to promote screening and prevention. Low-resourced settings suffer from many similar challenges to digital technology use as in high-income nations, however, these are more severely evident, most notably the lack of access to internet, mobile connectivity and electricity. Additional barriers and facilitators unique to settings in SSA were also found, including sociocultural values, beliefs, relevance and community preferences. These factors could act as significant drivers of success if adequately incorporated into mHealth tool design and implementation.

## Appendix 1

**TABLE 1-A1:** Mixed methods appraisal tool quality criteria.

**Outline of Criteria (41):**
**S: Screening Questions**
S.1-‘Are there clear research questions?’
S.2-‘Do the collected data allow to address the research questions?’
**1: Qualitative Studies**
1.1-‘Is the qualitative approach appropriate to answer the research question?’
1.2-‘Are the qualitative data collection methods adequate to address the research question?’
1.3-‘Are the findings adequately derived from the data?’
1.4-‘Is the interpretation of results sufficiently substantiated by data?’
1.5-‘Is there coherence between qualitative data sources, collection, analysis and interpretation?’
**4: Quantitative descriptive studies**
4.1-‘Is the sampling strategy relevant to address the research question?’
4.2-‘Is the sample representative of the target population?’
4.3-‘Are the measurements appropriate?’
4.4-‘Is the risk of nonresponse bias low?’
4.5-‘Is the statistical analysis appropriate to answer the research question?’
**5: Mixed methods studies**
5.1-‘Is there an adequate rationale for using a mixed methods design to address the research question?’
5.2-‘Are the different components of the study effectively integrated to answer the research question?’
5.3-‘Are the outputs of the integration of qualitative and quantitative components adequately interpreted?’
5.4-‘Are divergences and inconsistencies between quantitative and qualitative results adequately addressed?’
5.5-‘Do the different components of the study adhere to the quality criteria of each tradition of the methods involved?’

*Source*: Hong QN, Fàbregues S, Bartlett G, et al. The Mixed Methods Appraisal Tool (MMAT) version 2018 for information professionals and researchers. Educ Inf. 2018;34(4):285–291. https://doi.org/10.3233/EFI-180221

## Appendix 2

**TABLE 1-A2:** Assessing the quality of included studies using the mixed methods appraisal tool.

Author (year)	MMAT criteria																
	Screening		Qualitative studies					Quantitative studies					Mixed methods studies				
	S.1	S.2	1.1	1.2	1.3	1.4	1.5	4.1	4.2	4.3	4.4	4.5	5.1	5.2	5.3	5.4	5.5
Salako et al.^30^	N	Y	-	-	-	-	-	Y	CT	Y	N	N	-	-	-	-	-
Caster et al.^32^	Y	Y	-	-	-	-	-	Y	Y	CT	Y	Y		-	-	-	-
Bonful et al.^31^	Y	Y	Y	Y	Y	Y	Y	Y	N	Y	Y	Y	Y	Y	Y	Y	Y
Laker-Oketta et al.^33^	Y	Y	-	-	-	-	-	Y	Y	CT	Y	Y	-	-	-	-	-
Kabukye et al.^34^	Y	Y	Y	Y	Y	Y	Y	Y	N	CT	N	Y	Y	N	N	CT	N

*Source:* Hong QN, Fàbregues S, Bartlett G, et al. The Mixed Methods Appraisal Tool (MMAT) version 2018 for information professionals and researchers. Educ Inf. 2018;34(4):285–291. https://doi.org/10.3233/EFI-180221

Y, yes; N, no; CT, Can’t tell; MMAT, mixed methods apraisal tool.
